# Morphological and Functional Outcomes after Intravitreal Dexamethasone Injection for Macular Edema in Patients with Central Vein Occlusion at 48-Week Follow-Up

**DOI:** 10.1155/2020/6830148

**Published:** 2020-02-11

**Authors:** Cristina Nicula, Dorin Nicula, Anca Rednik, Adriana Bulboaca, Ovidiu Crișan

**Affiliations:** ^1^University of Medicine and Pharmacy “Iuliu Hațieganu”, Faculty of Medicine, Department of Ophthalmology, Cluj-Napoca, Romania; ^2^Oculens Clinic, Cluj-Napoca, Romania; ^3^Emergency County Eye Hospital, Cluj-Napoca, Romania; ^4^University of Medicine and Pharmacy “Iuliu Hațieganu”, Faculty of Medicine, Department of Physiopathology, Cluj-Napoca, Romania; ^5^University of Medicine and Pharmacy “Iuliu Hațieganu”, Faculty of Pharmacy, Department of Organic Chemistry, Cluj-Napoca, Romania

## Abstract

**Purpose:**

The purpose of the study was to assess the efficacy of intravitreal dexamethasone implant (IDI: Ozurdex®) injection in eyes with macular edema due to retinal vein occlusion. *Material and Method*. A retrospective, nonrandomized study was conducted in patients with macular edema (ME) due to retinal vein occlusion (RVO) who undertook intravitreal Ozurdex® as first-line treatment. We performed a complete ocular exam including macular OCT.

**Results:**

The mean BCVA (logMar) improved from 0.420.42 ± 0.23 logMar at baseline to 0.21 ± 0.23 logMar at 48 weeks in the BRVO group and from 0.72 ± 0.16 logMar at baseline to 0.31 ± 0.23 logMar at 48 weeks in the CRVO group. In both groups, CFT values decreased significantly compared to baseline (*p* < 0.0001 at each timepoint). Reinjection for recurrent macular edema after 18 weeks was indicated in five eyes (41.67%) in the BRVO group and in six eyes (25%) in the CRVO group. Cataract developed in two eyes (16.67%) in the BRVO group and in one eye (4.17%) in the CRVO group. The IOP was higher than 25 mmHg in two cases in the BRVO group (16.66%) and in three cases (8.33%) in the CRVO group.

**Conclusion:**

Ozurdex® injected intravitreally significantly improved the mean CFT and BCVA in eyes with macular edema due to retinal vein occlusion.

## 1. Introduction

Central retinal vein occlusion (CRVO) and branch retinal vein occlusion (BRVO) are between the most significant causes of decreased visual acuity due to the existence of macular edema (ME), whether the fovea is perfused or not [1]. ME is the result of increased intraluminal pressure, vascular endothelial damage, and impaired blood-retina barrier that results in leakage, relative ischemia, and low-grade inflammation [2]. For many years, the standard procedure for patients with ME has been grid laser photocoagulation [3, 4]. The Central Vein Occlusion Study not only confirmed its beneficial effects on ME but also showed that there was no statistical significant difference in visual acuity [5]. Over the last decade, the therapeutic options for ME associated with retinal vein occlusion (RVO) were revolutionized by intravitreal pharmacotherapy. Data revealed by clinical studies regarding treatment of ME due to retinal vein occlusion with intravitreal injection with antivascular endothelial growth factor (VEGF) and dexamethasone showed substantial morphological and functional improvements in comparison to those obtained by laser therapy alone [1]. Ozurdex® was developed as a biodegradable vehicle for dexamethasone administered by intravitreal implant, which delivers a 700 *μ*g dose of this drug to the retina and the vitreous. It was approved for use in the treatment of RVO in the United States of America (USA), Europe, and Switzerland. Several studies showed that intravitreal steroid injections have anti-inflammatory, antiangiogenic, and antivascular permeability characteristics and are effective for treating RVO-related ME [1, 2, 6–8].

Therefore, the purpose of the study was to evaluate the efficacy of intravitreal dexamethasone implant (IDI: Ozurdex®) injection in eyes with macular edema due to retinal vein occlusion.

## 2. Materials and Methods

### 2.1. Study Design

A retrospective, nonrandomized study was performed, based on the medical records of patients who had macular edema (ME) consequently to retinal vein occlusion (RVO) and had been treated as first-line treatment with Ozurdex intravitreal injection between September 2015 and December 2017 in Oculens Clinic, Cluj-Napoca, Romania. The study began after obtaining approval from the Clinical Ethics Committee.

### 2.2. Subjects

Newly diagnosed naïve RVO patients who had macular edema  under 3 months at first presentation with a baseline central foveae thickness (CFT) of >300*μ* and visual acuity of +0.3 logarithm of the minimum angle of resolution (logMar) or worse were included. The exclusion criteria were coexisting retinal disease (such as diabetic retinopathy, age related macular degeneration, vitreomacular traction, or epiretinal membrane), or media opacities (cataract) that could decrease visual acuity (VA), and pregnancy. Patients who had previously received treatment for ME (anti-VEGF, steroids, and laser), with a history of ocular surgery (except cataract) and trauma, were excluded. All patients underwent standardized examination including measurement of best-corrected visual acuity (BCVA) using a projection chart at 5 m, slit-lamp biomicroscopy, fundus examination using a postdilation +90 diopter lens and a three mirror contact lens, measurement of intraocular pressure (IOP) via applanation tonometry, and color fundus photography. Fluorescein angiography (FA) (HRA-2; Heidelberg Engineering, Heidelberg, Germany) and optical coherence tomography (OCT) imaging (Triton, Topcon, Japan) of the macula were performed prior to treatment initiation. At each visit, the aforementioned examinations were performed, with exception of FA. Macular optical coherence tomography (OCT) was used to measure central foveae thickness (CFT), which was defined as mean thickness of the neurosensory retina in central 1 mm diameter region, and was computed via OCT mapping software provided with the device. Fluorescein angiography was performed in order to establish capillary dropout zones at the fovea and peripheral retina, and for leakage, as causes of ME.

Written informed consent for treatment was obtained from all patients, and the study complied with the principles of the Declaration of Helsinki (1964) and its amendments (Tokyo 1975, Venice 1983, and Hong Kong 1989).

### 2.3. Study Protocol

All injections were performed under sterile conditions in the operating room, after application of topical anesthesia (Benoxi–Oxybuprocaini Hydrochloridum, Unimed Pharma LTD., Slovakia) and of 10% povidone-iodine solution (Betadine Egis Pharmaceuticals PLC, Hungary); scrub was used on the lids and lashes, and 5% povidone-iodine was administered in the conjunctival sac. Intravitreal Ozurdex® 0.7 mg (Ozurdex®, Allergan Inc., Irvine, CA, USA) was injected through the pars plana into the vitreous, at 3.5 mm posterior to the limbus with a customized, single-use 22-gauge applicator. After the injection, each patient was prescribed steroids and antibiotics five times a day for one week. Patients were instructed to return to the hospital if they experienced decreased vision, eye pain, or any new symptoms.

### 2.4. Safety Evaluation

All the patients were followed up for 48 weeks. During the study period, the patients were monitored for adverse effects (IOP measurement; lens transparency). In the first year, the patients were examined the day after injection and 4 weeks, 8 weeks, 12 weeks, 24 weeks, and 48 weeks after injection.

Panretinal or sectorial photocoagulation was applied to the patients who showed any kind of neovascularization during the follow-up. Panretinal photocoagulation was applied to the CRVO patients who showed neovascularization of the iris or on optic disc. Sectorial laser photocoagulation was applied to the BRVO patients who showed any kind of neovascularization, and the treatment area covered the entire ischemic area that was detected via FA.

### 2.5. Data Collection

Data collected from patients' records included age, gender, type of RVO, ischemic status, types of RVO, associated risk factors, complications after injection, BCVA (converted to logarithm of the minimum angle of resolution, logMar), intraocular pressure, and CFT. Visual acuity and CFT were measured at all timepoints (baseline, 4 weeks, 8 weeks, 12 weeks, and 48 weeks).

### 2.6. Statistical Analysis

Visual acuity and the CFT values between baseline and the other timepoints were assessed with repeated measurement tests. Categorical variables were compared using the chi-square test. A *p* value <0.05 was considered statistically significant.

## 3. Results

### 3.1. Demographics

Thirty-six eyes of 36 patients were included. The average age of the patients was 59.33 ± 15.74 years (range 22–89). Twenty-four of the patients (66.67%) had nonischemic CRVO, while 12 (33.33%) had nonischemic BRVO and received IDI injection as the first-line treatment for ME. No significant difference was seen between the two groups with respect to age and gender (*p*=0.33). The follow-up period was 48 weeks. Demographic characteristics of the patients are shown in Table 1.

Systemic comorbidities included diabetes mellitus in 9% of the patients, atherosclerosis (9%), ischemic heart disease (9%), and hypertension (19%). Primary open angle glaucoma was present in two cases of BRVO (16.66%) and in four cases of CRVO (16.66%). All these patients followed a topical treatment with fixed combination (timolol 0.5% and dorzolamide) or prostaglandin analogue, with well-controlled IOP. Small hyperopia was present in 16 cases (44.44%).

**Figure 1:**
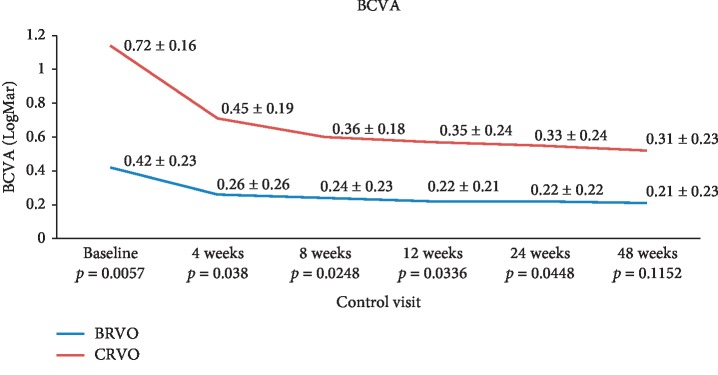
Mean BCVA values from baseline to follow-up visits after intravitreal dexamethasone injection in the two groups and the *p* values between the groups.

In the CRVO group, the mean BCVA (logMar) value was 0.72 ± 0.16 logMar at baseline and improved to 0.45 ± 0.19 logMar after 4 weeks, 0.36 ± 0.18 logMar after 8 weeks, 0.35 ± 0.24 logMar after 12 weeks, 0.33 ± 0.24 logMar after 24 weeks, and 0.31 ± 0.23 logMar at 48 weeks (see Figure 1). In the CRVO group, the difference between the baseline and postinjection follow-up BCVA values was statistically significant. BCVA values at each control visit improved significantly compared to baseline (*p*=0.0012 after 4 weeks;*p* < 0.0001 at 8 weeks, 12 weeks, 24 weeks, and 48 weeks).

In the BRVO group, the mean BCVA (logMar) value was 0.42 ± 0.23 logMar at baseline and improved to 0.26 ± 0.26 logMar after 4 weeks, 0.24 ± 0.23 logMar after 8 weeks, 0.22 ± 0.21 logMar after 12 weeks, 0.22 ± 0.22 logMar after 24 weeks, and 0.21 ± 0.23 logMar at 48 weeks (see Figure 1). In the BRVO group the difference between the baseline and postinjection follow-up BCVA values was statistically significant. BCVA values at each control visit improved significantly compared to baseline (*p*=0.0017 after 4 weeks; <0.0001 at 8 weeks, 12 weeks, 24 weeks, and 48 weeks).

The difference between the two groups regarding BCVA was statistically significant at baseline (*p*=0.0057), at 4 weeks (*p*=0.038), at 8 weeks (*p*=0.0248), at 12 weeks (*p*=0.0336), and at 24 weeks (*p*=0.0448), but there was no statistically significant difference at 48 weeks (*p*=0.1152) (see Figure 1).

**Figure 2:**
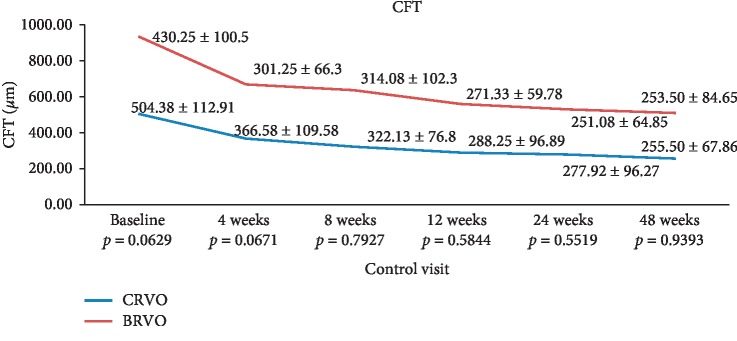
The difference between the baseline and postinjection follow-up CFT values in both groups and *p* values between the groups.

In the CRVO group, the mean CFT value was 504.38 ± 112.91 *μ*m at baseline and decreased to 366.58 ± 109.58 *μ*m after 4 weeks, 322.13 ± 76.80 *μ*m after 8 weeks, 288.25 ± 96.89 *μ*m after 12 weeks, 277.92 ± 96.27 *μ*m after 24 weeks, and 255.50 ± 67.86 at 48 weeks (see Figure 2). CFT values at each control visit improved significantly compared with baseline CFT values (*p* < 0.0001 at each timepoint).

In the BRVO group, the mean CFT value was 430.25 ± 100.5 *μ*m at baseline and decreased to 301.25 ± 66.30 *μ*m after 4 weeks, 314.08 ± 102.30 *μ*m after 8 weeks, 271.33 ± 59.78 *μ*m after 12 weeks, 251.08 ± 64.85 *μ*m after 24 weeks, and 250.80 ± 84.65 after 48 weeks (see Figure 2). CFT values at each control visit improved significantly compared with baseline CFT values (*p*=0.012 at 4 weeks, *p*=0.0103 at 8 weeks, *p* < 0.0001 at 12 weeks, 24 weeks, and 48 weeks).

The difference between the two groups regarding CFT values was not statistically significant at any control visits (*p*=0.0629 at baseline visit, *p*=0.0671 at 4 weeks, *p*=0.7929 at 8 weeks, *p*=0.5844 at 12 weeks, *p*=0.5519 at 24 weeks, and *p*=0.9393 at 48 weeks) (see Figure 2).

Reinjection for recurrence of CFT elevation demonstrated by the macular OCT at 18 weeks was indicated in 6 cases (25%) in the CRVO group and in 5 cases in the BRVO group (41.67%). These cases presented for a check-up at 18 weeks (even it was not the check-up timepoint) because they observed a significantly visual acuity decrease. These cases were treated using a second injection of anti-VEGF such as bevacizumab (Avastin). The switch had a good rationale due to the different mode of action of these agents (Ozurdex versus Becacizumab) and also because of financial reasons. In Romania, the intravitreal injection with Ozurdex is not covered by the National Health Care System.

Intraocular pressure was measured in both groups in the first week after the injection. In the CRVO group and the BRVO group, the mean IOP value in the first week was 19.08 ± 2.95 mmHg and 18.75 ± 2.90 mmHg, respectively. The IOP was higher than 25 mmHg in three cases (8.33%) in the CRVO group and in two cases (16.66%) in the BRVO group two months after the intravitreal injection. IOP higher than 10 mmHg was present in 2 eyes (4.8%) in the CRVO group and in one eye (1.2%) in the BRVO group. Topical antiglaucomatous drugs were required in all these cases. Topical timolol 0.5% combined with dorzolamide in fixed combination was administered twice per day. Moreover, no statistical significant difference was shown between IOP values in the third and fourth month and baseline values (*p*=0.332 in the CRVO group and *p*=0.673 in the BRVO group). One patient required surgical treatment such as trabeculectomy.

Cataract developed in one eye (4.17%) in the CRVO group and in two eyes (16.67%) in the BRVO group and required phacoemulsification with intraocular artificial lens implantation. Conjunctival hemorrhages occurred in five patients (13.8%). None of the patients developed endophthalmitis, vitreous hemorrhage, or retinal detachment.

## 4. Discussions

Ozurdex® (dexamethasone intravitreal implant) is an intravitreal implant containing 0.7 mg (700 *μ*g) dexamethasone in the Novadur solid polymer drug delivery system (Allergan Inc., Irvine, CA, USA). It is a potent corticosteroid, which suppresses inflammation by inhibiting multiple inflammatory cytokines resulting in decreased edema, fibrin deposition, capillary leakage, and migration of inflammatory cells [9]. The National Institute for Health and Care Excellence recommends the dexamethasone 0.7 mg intravitreal implant as an option for the treatment of ME following CRVO and BCVO when treatment with laser photocoagulation has not been beneficial or was not considered because of the extent of the hemorrhage [10]. The rationale for the use of steroids for ME is that steroids lessen retinal capillary permeability and stop the expression of the VEGF gene and the metabolic pathway of VEGF.

Demographic data from our study were similar with those showed in a recent study [11], which evaluated the efficacy and safety of intravitreal steroids for ME secondary to retinal vein occlusion.

Functional results from our study showed a statistical significant improvement in BCVA in both groups at each timepoint comparing with baseline (*p* < 0.005). The results were similar to those of previous studies. In the GENEVA study, the improvement in BCVA by 15 letters or more was 29% at 60 days and 22% at 180 days [4, 12]. In the COBALT study, 65% of the patients gained more than 15 letters at the EDTRS logMar chart at 6 months and 56% at 12 months [13]. The results of recent studies revealed that treatment of BRVO as early as 2 weeks after onset of ME enhanced visual outcomes [14]. In the SOLO study, the improvement in BCVA in the BRVO group was from 0.6 to 0.45 logMar after 24 weeks after the treatment, and in the CRVO group, VA increased from 0.7 to 0.52 logMar after 24 weeks [15]. Simsek et al. [10] showed in their study that BCVA improved significantly compared with baseline (*p*=<0.001) after the second injection of Ozurdex intravitreal implant. Mayer et al. [16] demonstrated an improvement in BCVA by 6.6 ± 1.7 letters in the CRVO group and by 7.8 ± 2.9 letters in the BRVO group.

Mean reduction in central macular thickness in our study was significant in both groups at each time point (*p* < 0.005) and was comparable with other studies. In the GENEVA study, it was shown a mean reduction of 119 *μ*m at 180 days following treatment [13]. Singer et al. [17] showed a reduction in CFT of 195 *μ*m. Shahina et al. [9] demonstrated in their study a mean reduction in CFT of 181.3 ± 210.92 *μ*m. Simsek et al. [10] showed a statistically significant improvement (*p* < 0.001) of CFT 4 months after intravitreal dexamethasone injection. Moreover, they observed the recurrence of CFT elevation in 65.3% of patients in the BRVO group and in 68.1% in the CRVO group 4 months after the second injection of intravitreal dexamethasone implant. In the present study, the recurrence after the first injection was present in 41.67% patients with BRVO and in 25% patients with CRVO. Bezatis et al. [11] reported that the mean CFT maintained was significantly reduced (*p* < 0.001) compared with the baseline at each follow-up visit.

In the current study, the recurrence of ME appeared in six eyes (25%) in the CRVO group and in five eyes (41.67%) in the BRVO group at 4.5 months from baseline. Published reports in which reinjections have been made after shorter intervals on an “as needed” basis are now available [18, 19]. In an earlier published retrospective assessment of 33 RVO-afflicted eyes, retreatment with dexamethasone was necessary at 4.7 ± 1.1 months after the first injection and at 5.1 ± 1.5 months after the second one in order to sustain a significant improvement in the best-corrected visual acuity and in the central retinal thickness [16]. Considering the results of the aforementioned studies, it is clear that the effects of intravitreal-administered dexamethasone can be sustained for 4 months (range: 3 to 7 months) irrespective of the patient's clinical background. A retreatment initiation on an “as needed” basis would require injection intervals of substantially less than 6 months for the vast majority of eyes [6, 15, 18, 20]. Moreover, frequent and repeated treatments with Ozurdex enlarge the risk of ocular side effects such as raised IOP and cataract formation. That is why in our study we preferred to use as a second injection an anti-VEGF medication.

In this study, cataract that decreased VA appeared in one eye (4.17%) in the CRVO group patients and in two eyes (16.67%) in the BRVO group during the follow-up. The results are similar with others studies [17]. Cataract may form because of long-term steroid secretion after single injection. The risk for cataract is higher after two injections of dexamethasone intravitreal implant [21]. Ozkaya et al. [8] reported a rate of cataract of 4.4% after a single intravitreal dexamethasone injection. The COBALT study showed a progression in lens opacities in 36% of patients [13]. Mayer et al. [16] reported a rate of 50% of eyes with cataract after three Ozurdex injections. Reid et al. [22] showed that the risk of cataract formation is higher in patients receiving multiple IDI injections. Nevertheless, cataract may form because of long-term steroid secretion after a single injection [10]. Meyer and Schönfeld did not reveal any cataract progression at 6 months after intravitreal injection of Ozurdex [23]. There are some conflicting studies that reported no cataract progression even after accidental intralenticular Ozurdex implant administration [24, 25]. In addition, many authors have revealed a resolution of the ME with an intralenticular implant [24, 26–29].

In our study, intraocular pressure increased in three eyes (8.33%) in the CRVO group and in two eyes (16.66%) in the BRVO group. In 2 eyes in the CRVO group, the IOP increased more than 10 mmHg. These results were lower in comparison to those revealed by Schmitz et al. [19]. In their retrospective study on 342 retinal vein occlusions, the IOP increased in 20% afflicted eyes after intravitreal injections of dexamethasone. In the Shasta trial [30], in 32.6% of the CRVO- and BRVO-afflicted eyes, an IOP increase of  ≥10 mm Hg was reported. Intraocular pressure-lowering medication was given in 29.1% of the patients, while in 1.7%, incisional glaucoma surgery was performed. Mayer and Schönfeld [23] described an elevated IOP (>5 mmHg) in 40% of patients. Joshi et al. [31] reported an increase in IOP in 27% of the eyes, which needed to be medically controlled. In the GENEVA study [7], the authors showed an elevation of 25% of IOP at 6 months after intravitreal injection. On the contrary, Meyer and Schönfeld [23] did not notice any increase in IOP 6 months after the treatment. The increased IOP after Ozurdex intravitreal injection appears as a result of the steroid intravitreal injection. Dot et al. showed that steroid-induced glaucoma is the most common side effect associated with the dexamethasone intravitreal injection. We believe that each patient from our study who developed ocular hypertension was steroid responder [32]. Several pathogenetical mechanisms have been proposed for steroid-induced IOP elevation as a result from biochemical and structural changes in the trabecular meshwork (TM). Inhibition of extracellular matrix material degradation with the accumulation of fibronectin, glycosaminoglycan, laminin, and elastin in the TM, reduced phagocytotic capacity, decreased activity of protease, increased DNA content and nuclear size, reorganization of the TM cytoskeleton (which is unclear), formation of intercellular junctions, and rearrangement of specific protein synthesis are the main effects of steroids on the TM activity http://ghrnet.org/index.php/IJOR/article/view/2513/2894 [33–36]. François [37] and Armaly [38] suggested that the increased IOP is due to the alteration of the mucopolysaccarides, leading to their accumulation in the TM. Experimental studies have reported that steroids significantly increase expression of different genes in human TM [39–41].

In our study, we did not have any endophthalmitis after Ozurdex intravitreal implantation. The results are similar with previous studies [42, 43].

In our study, one eye (8.33%) with BRVO received a sectorial photocoagulation after 12 weeks and three eyes (12.5%) with CRVO received a panretinal laser photocoagulation 12 weeks after injection. In these cases, 12 weeks after the intravitreal treatment, the patients developed new vessels on the optic disc as a sign of ischemic form of retinal vein obstruction, even if they had a nonischemic form at the beginning of the study. The goal of the treatment was to decrease neovascular changes and prevent the development of neovascular glaucoma. There are studies that revealed that 30% of eyes with nonischemic CRVO at first may convert to ischemic type [44–47]. Trombosis of the retinal veins give rise to an increase in retinal capillary pressure with a higher capillary permeability and leakage of fluid and blood into the retina. Once the ischemia appears, the production of vascular endothelial growth factor (VEGF) is facilitated and promotes the retinal capillary permeability and leakage into the extracellular space ending in development of ME [48].

The present study has some limitation regarding the small sample size, the short period of follow-up and the absence of a control group. To our knowledge, this is the first Romanian study regarding the efficacy of intravitreal Ozurdex injection for ME after retinal vein occlusion. Nonetheless, further studies with active controls are needed to completely understand the efficacy and safety of intravitreal dexamethasone implant injection.

## 5. Conclusions

Intravitreal Ozurdex® injection significantly improved mean BCVA and reduced CFT in eyes with macular edema due to retinal vein occlusion. The treatment is safe and effective. Cataract formation and increasing IOP demands regular visits in patients treated with intravitreal Ozurdex.

## Figures and Tables

**Table 1:** Demographic characteristics of patients included in the study.

Present pathology	Mean age	Males (%)	Females (%)
CRVO	61.17 ± 15.43	66.67	33.33
BRVO	55.67 ± 16.37	50	50

## Data Availability

The data used to support the findings of this study are available from the corresponding author upon request.
